# Public knowledge and attitudes towards HIV and people with HIV in Switzerland: results of a national survey

**DOI:** 10.1186/s12889-026-27629-1

**Published:** 2026-05-18

**Authors:** Thomas Grabinger, David Haerry, David Jackson-Perry, Marlon Gattiker, Corinna Oberle, Dominique L. Braun, Katharine E. A. Darling

**Affiliations:** 1 Gilead Sciences Switzerland Sàrl, Zug, Switzerland; 2 Positive Council Switzerland, Zurich, Switzerland; 3 Infectious Diseases Service, Lausanne University Hospital, Lausanne, Switzerland; 4 Swiss AIDS Federation, Zurich, Switzerland; 5 Institute for Infectious Diseases, Clinic Hirslanden Zurich, Zurich, Switzerland; 6 Department of Infectious Diseases and Hospital Epidemiology, University Hospital Zurich, University of Zurich, Zurich, Switzerland; 7 Independent Medical Writer, Lausanne, Switzerland

**Keywords:** HIV knowledge, Public knowledge, Attitudes, People living with HIV

## Abstract

**Background:**

HIV-stigma impacts negatively on the quality of life of people with HIV (PWH) and can constitute a barrier to HIV prevention services, diagnosis and retention in care. Knowledge that HIV treated with effective antiretroviral therapy is a non-fatal chronic condition and awareness of the U = U (undetectable equals untransmittable) message can mediate stigmatising attitudes. We conducted a survey to assess public knowledge and attitudes regarding HIV and PWH in Switzerland.

**Methods:**

Members of the public aged ≥ 18 years were randomly selected by a panel institute providing representative population surveys and quota sampling across demographic variables including gender, age, canton of residence and educational level. Participants were invited to complete a questionnaire with closed-ended questions via computer-assisted telephone and/or web interviews.

**Results:**

A total of 1015 participants (50.7% women, 49.1% men, 0.2% diverse) completed the survey. While 76% stated they felt informed about HIV, misconceptions regarding HIV-acquisition risk were reported, including kissing (22%), insect bites (13%), and shared use of everyday objects (8%). Over half (55%) cited journalistic media as their source of HIV knowledge. Regarding the U = U message, 22% believed it to be true, 22% believed it to be untrue and 56% did not know. Awareness of U = U was higher in younger people and those with higher education. The majority had low (90%) or moderate (9%) HIV-stigma sum scores; higher scores were associated with lower education. In total, 12.6% had heard of pre-exposure prophylaxis (PrEP), of whom 54.7% identified the correct PrEP description.

**Conclusions:**

Although 76% of participants felt informed about HIV, only 22% agreed with the U = U message. Misconceptions regarding HIV acquisition risk were observed. High HIV-stigma scores were observed among a minority of participants and were associated with lower education. Tailored awareness campaigns could enhance HIV knowledge, disseminate the U = U message and address societal HIV-stigma.

**Supplementary Information:**

The online version contains supplementary material available at 10.1186/s12889-026-27629-1.

## Introduction

Since the HIV/AIDS pandemic began in the 1980 s, medical advances have made possible HIV diagnosis and treatment. Effective antiretroviral therapy (ART) has shifted HIV from being a fatal infection to a chronic condition compatible with a near-normal life expectancy.

The ‘Swiss Statement’ in 2008 [1] and the undetectable equals untransmittable (U = U) campaign, launched in 2016 [2], have provided a framework to reduce the fear of HIV acquisition based on the scientific consensus that when adherent use of ART suppresses plasma HIV RNA to undetectable levels in people with HIV (PWH), there is no risk of HIV acquisition through sex [3, 4]. Despite endorsement of U = U by multinational organisations including the World Health Organisation, the Joint United Nations Programme on HIV/AIDS and the Centers for Disease Control and Prevention, wider dissemination of the U = U message is incomplete [5, 6]. In healthcare settings, lack of knowledge of such HIV prevention messages is associated with stigmatising attitudes towards PWH [7]. Thus, while PWH on ART live physically well, HIV-stigma persists.

HIV-stigma can be considered in terms of internalised, anticipated and enacted constructs, which describe how PWH experience stigma [8]. Another way to consider HIV-stigma is in terms of populations and settings. Healthcare settings can perpetuate HIV-stigma by not reviewing outdated ‘precautionary’ measures adopted for PWH, such as wearing double gloves, placing ‘HIV + ’ stickers on medical notes, and placing PWH at the end of operating lists [8]. Stigmatising attitudes may also be encountered in the general public, together with unfounded fear and lack of up to date HIV knowledge, including of the U = U message [9].

HIV-stigma has been explored among PWH and in healthcare settings in Switzerland [10–12] and the rest of Europe [7, 13]. In Switzerland, an estimated 18,000 people (range 15,000—20,000) are living with HIV [14] and the HIV incidence in 2023 was 4 new cases per 100,000 inhabitants [15]. Pre-exposure prophylaxis (PrEP) to prevent HIV transmission is available through a national programme, SwissPrEPared, which was launched in April 2019 [16]. The SwissPrEPared programme ensures standardisation of PrEP counselling and screening for sexually transmitted infections across providers in the three main linguistic regions of Switzerland (French-, German-, and Italian-speaking) [17]. By the end of 2023, 5750 people were on PrEP in Switzerland, mostly men who have sex with men [18].

In the current survey, we aimed to explore HIV knowledge and attitudes towards HIV and PWH among members of the public in Switzerland.

## Methods

### Ethics statement

As defined under a decree of Swissethics (Version 1.0, 01.02.2020), the umbrella organisation coordinating Swiss cantonal ethics committees, quality assurance projects are not subject to ethics committee approval or waiver requirement provided they do not aim to generate new generalisable medical knowledge and do not involve the collection of biological material or the processing of identifiable health‑related personal data [19]. Formal ethics approval for this survey was therefore not required. The survey was nonetheless conducted in accordance with relevant national guidelines and regulations, including the principles of the Declaration of Helsinki.

### Patient and public involvement

This study was set up as a quality assurance project in accordance with the Swiss Ethics Committee on research involving humans. Community representatives living with HIV and affiliated to different organisations were involved throughout the study process, from study design to manuscript review.

### Survey setting

The survey was developed in collaboration with experts in the field of healthcare sustainability, in accordance with the CHERRIES checklist [20] (Supplementary Material S1), and was conducted in October 2023. Participants aged ≥ 18 years old were recruited by a panel institute providing representative population surveys (Bilendi, www.bilendi.ch). Participants were selected randomly from the Bilendi pool, using representative quota sampling by gender, age, canton of residence and educational level, until quotas were reached. Participants were invited to answer questions anonymously via computer-assisted telephone and/or web interviews. Reasons for non-participation or survey discontinuation were not asked. Participation was reimbursed with a token sum of CHF1.60 (equivalent to £1.50). Bilendi had no access to the survey content; a partner institute (Medupha, www.medupha.com) was responsible for database hosting, survey programming and survey content analysis.

### Sample size calculation

At the time of the survey, Switzerland had an estimated population of 8.8 million. The sample size was calculated based on a 95% confidence interval (Z-score of Z = 1.96) and a 3% margin of error (E = 0.03) using the standard formula for sample size estimation: n = Z^2^ p (1—p)/E^2^, with p representing the true underlying proportion of the population (the fraction of individuals in the target population who have the characteristic or outcome of interest). A proportion of *p* = 0.5, assumes maximum uncertainty and ensures maximum variability, the most conservative estimate scenario. This resulted in a finite population sample size of 1067; an adjusted sample size, calculated upon applying the finite population correction, remained unchanged owing to the large population size. With a final sample size of 1015 individuals, slightly below the target of 1067, the margin of error increased modestly, from 3% to 3.08%

### Survey questionnaire (Supplementary material S2)

The questionnaire used for a similar survey in Austria was employed, translated into French by a certified translation agency, and with some adjustments to make it more specific to Switzerland. The questionnaire adjustments were based on discussion with and input from an expert panel consisting of community representatives, patient advocates, and healthcare professionals. Adjustments included improved adherence to the UNAIDS terminology guidelines [21] and modifying the responses to the question on sources of information on HIV or AIDS to better reflect the Swiss landscape. Specific questions to assess knowledge on U = U and pre-exposure prophylaxis (PrEP) were added, and the medical conditions section was modified to include questions on syphilis, chlamydia and hepatitis C.

The questionnaire gathered data on participant knowledge and attitudes regarding medical conditions, including HIV, and attitudes towards PWH. The questionnaire items were aligned with the UNAIDS global AIDS monitoring indicators for knowledge about HIV prevention and discriminatory attitudes towards people living with HIV [22]. Throughout the questionnaire, the terms, ‘HIV’ and ‘HIV/AIDS’ were used interchangeably in case participants were not familiar with the difference. At the end of the survey, participants were shown a brief educational text providing information about HIV, including the difference between HIV and AIDS, HIV acquisition risk, U = U, PrEP, and the near-normal life-expectancy for PWH in Switzerland on ART. Although the questionnaire was not translated into Italian, persons living in the Italian-speaking canton of Ticino were not screened out of the survey.

For quality assurance, incomplete questionnaires, questionnaires completed in under three minutes (‘speeders’), and those with contradictory responses upon quality checking were excluded from analysis.

### Participant demography

The following demographic data were collected from participants completing the questionnaire: gender, age, canton of residence in Switzerland, language (French or German), type of residence (urban, semi-urban or rural), education level, employment category (in training, employed, unemployed or retired), and approximate monthly income after tax and social security contributions. As the invitation to participate was sent out randomly to individuals from the quality-assured online panel according to predefined quotas based on age, gender, region, and level of education, it was not possible to collect information regarding the characteristics of non-responders nor potential reasons for refusing or not being able to participate.

### Knowledge and attitudes regarding different medical conditions

Prior to focusing on HIV, participants were asked how well-informed they felt they were about different medical conditions (diabetes, cancer, HIV/AIDS, Coronavirus/COVID-19, hypertension, syphilis or chlamydia, and hepatitis C) and whether they considered these conditions to be life-threatening. Responses were formulated as a four-point Likert-like scale with a ‘don’t know’ option. Participants were then asked to rate whether there had been medical advances in the treatment of each of these conditions in the last ten years, with response options ranging from 1 (no progress) to 10 (significant progress).

### Focus on HIV: information sources and personal experience

Following the health-related issues section, all questions focused on HIV. Participants who had indicated some level of knowledge about HIV/AIDS were asked to indicate their source of information from a list of options (family/friends, sexual partners, sex education, healthcare professionals, public health campaigns, social media and journalistic media; multiple responses possible). They were asked whether information they had seen or heard in the media in the preceding six months had been positive or negative towards PWH (Likert-like scale), whether they personally knew anyone living with HIV (themselves, family members, friends, acquaintances, other; multiple responses possible), and whether they personally had ever had an HIV test.

### Knowledge about HIV acquisition risk and key treatment and prevention messages

Participants were asked if a range of exposures could result in HIV acquisition (response options: ‘true’, ‘not true’ and ‘don’t know’). They were then asked about U = U knowledge as follows: *If people with HIV have been on effective HIV therapy for at least 6 months (so the virus is no longer detectable in blood), they cannot transmit the virus to their partners through sex* (response options: ‘true’, ‘not true’, ‘don’t know’). They were also asked: *Thanks to modern HIV therapy, HIV is no longer a death sentence but a controllable chronic viral infection*, and *Thanks to effective HIV therapy (where the virus is no longer detectable in blood), women with HIV can now give birth to healthy children who are not HIV-positive*, with the same response options.

Finally, participants were asked if they had heard of PrEP. Those who had were invited to select the correct description of PrEP (four options: *emergency treatment after an HIV acquisition risk situation, a preventive medication that protects against HIV, a new, innovative therapy for people with HIV,* or *don’t know*).

### Attitudes towards PWH

Participants were asked: *To what extent would you say that people with HIV present a danger to society?* and, *To what extent do you believe that people with HIV or AIDS can engage in regular employment?* (four-point Likert-like scale plus option ‘don’t know’). They were then asked if they would mind a range of social/physical exposures to PWH, ranging from sitting next to someone with HIV to having sex (four-point Likert-like scale).

### Data analysis

Data are presented as n (%) for each parameter. Participants responding that they were living with HIV were retained in the analysis as patient-provider communication and patient education regarding HIV and U = U is neither uniform nor universal [5].

For the question on U = U, the answer ‘true’ was considered to represent U = U knowledge. Participants responding ‘true’ were examined by demographic parameters and in terms of HIV ‘experience’ (knowing any PWH, having been HIV-tested). Chi squared testing was performed to examine participant parameters against responses ‘true’ or ‘not true’. Responses to questions on attitudes towards PWH with a satisfactory Cronbach alpha coefficient, calculated to assess internal consistency of the questions, were used to develop an HIV-stigma sum score. Questions with unacceptable Cronbach alpha coefficients and ‘don’t know’ responses were excluded. To calculate HIV-stigma sum scores, stigmatising responses were assigned a score of 1 or 2, and all other responses a score of zero, giving a minimum HIV-stigma sum score of zero and a maximum score of 14 (Table 1). The mean, standard deviation and z-scores for each sum score were calculated. Z-score quartiles were taken to assign the sum scores to categories of low, moderate and high HIV-stigma. Non-parametric tests (Mann–Whitney-U and Kruskal–Wallis-H tests) were applied to examine HIV-stigma sum scores against participant parameters when distributions were not normal.

**Table 1:**
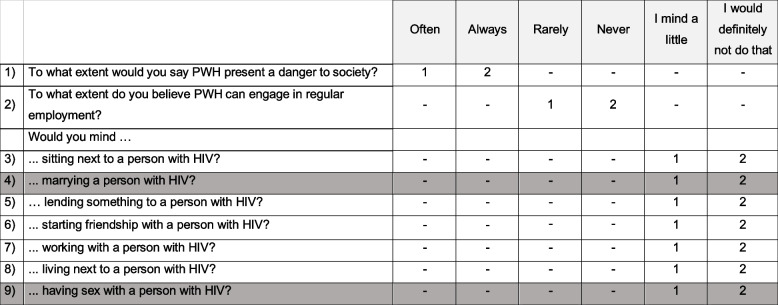
Scoring system for questions on attitudes towards people with HIV (PWH)

Questions 4) and 9) are shaded as these questions reduced the Cronbach alpha coefficient to below acceptable levels and were not included in the HIV-stigma sum score analysis

## Results

### Participants

In total, 1795 individuals were approached and questionnaires from 1015 participants were retained for analysis. Participant characteristics are shown in Table 2.

**Table 2:** Participant characteristics (*N* = 1,015)

	n (%)
Gender	
female	515 (50.7%)
male	498 (49.1%)
diverse	2 (0.2%)
Age groups	
18—29 years	168 (16.6%)
30—39 years	173 (17.1%)
40—49 years	175 (17.2%)
50—59 years	188 (18.5%)
60–69 years	183 (18.0%)
≥ 70 years	128 (12.6%)
Language	
German	721 (71.0%)
French	294 (29.0%)
Residence	
urban	351 (34.6%)
semi-urban	473 (46.6%)
rural	191 (18.8)
Education	
still in school/education	15 (1.5%)
compulsory school (primary and secondary level I)	158 (15.6%)
secondary level II^a^	470 (46.3%)
tertiary level^b^	358 (35.3%)
Employment	
student	52 (5.1%)
employed	643 (63.3%)
unemployed	82 (8.1%)
retired	228 (22.5%)
Monthly income	
< 2,000 CHF	88 (8.7%)
> 2,000–6,000 CHF	473 (46.6%)
> 6,000 CHF	280 (27.6%)
Informed about HIV/AIDS	
very well informed	177 (17.4%)
quite well informed	597 (58.8%)
Knowing a person with HIV/AIDS	
yes	258 (25.4%)
Ever having been tested for HIV	
yes	459 (45.2%)

^a^secondary level II: baccalaureate schools, specialised secondary schools, vocational secondary schools, vocational schools, apprenticeships

^b^tertiary level: universities, universities of applied sciences, universities of teacher education, higher vocational education and training

### Questionnaire internal consistency

For the questions on attitudes towards PWH in Table 1, Cronbach’s alpha coefficient was 0.87, demonstrating satisfactory internal consistency. The Cronbach’s alpha coefficient fell when questions related to marrying or having sex with a person with HIV were included (Table 1).

### Knowledge and attitudes regarding different medical conditions

**Fig. 1:**
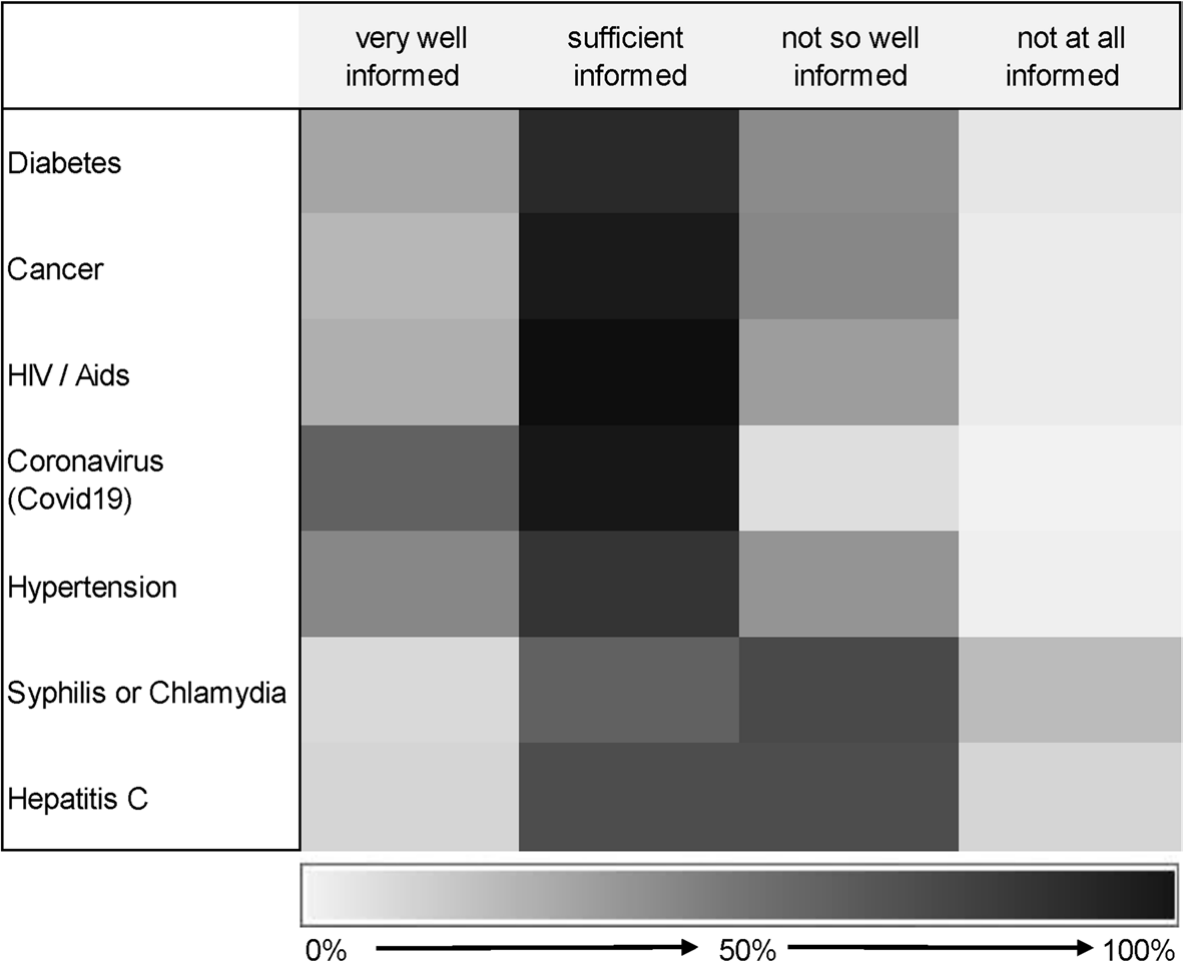
Heat map of how informed participants felt regarding different medical conditions (*N* = 1,015)

Overall, 83% (842/1015) of participants felt they were well-informed/quite well-informed about general conditions and 76.2% (774/1015) felt they were well-informed/quite well-informed about HIV/AIDS. Figure 1 shows how this compares to other conditions. Overall, 73.4% (745/1015) felt HIV was very/quite life-threatening. The median score for treatment advances in the preceding ten years (possible range 1—10) was 8 for HIV/AIDS and Coronavirus (COVID-19) and 7 for the other conditions.

### HIV information sources and personal experience

Regarding the sources of HIV information, the majority (55%, 542/1015) were informed by journalistic media (TV, newspaper and websites), while school/education (37%, 359/1015) and medical staff (35%, 348/1015) represented the main alternative sources (Supplementary material S3).

Considering items seen or read in the media during the preceding six months, 25.8% (262/1015) of participants had not seen anything in the media or did not remember. Of those who had, 55.4% (417/753) felt PWH were presented in an overall positive light, 38.2% (288/753) were neutral, and 6.4% (48/753) felt PWH were presented in a negative light. Regarding direct experience of HIV, 25.4% (259/1015) of participants knew someone with HIV, more frequently friends, colleagues and other acquaintances (5.9—13.2%) than partners or directly family members (0.7%, 7/1015). Six people (of 1015, 0.6%) stated they were living with HIV and 45.2% (459/1015) of participants had ever had an HIV test (Table 2).

### Knowledge about HIV acquisition risk and key treatment and prevention messages

**Fig. 2:**
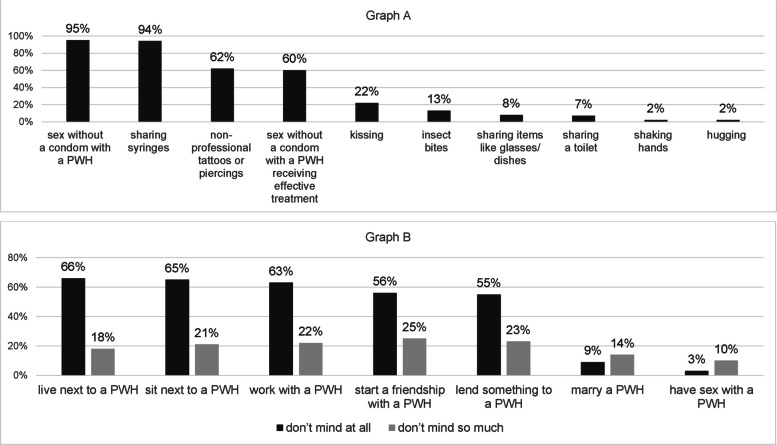
Knowledge about HIV acquisition risk and attitudes towards people with HIV (PWH). Graph A: Percentage of participants believing HIV acquisition can occur through different exposures. Graph B: Percentage of participants who responded, ‘don’t mind at all’ and ‘don’t mind so much’ to different social/physical contact scenarios

Regarding HIV acquisition risk, 94.7% (961/1015) believed acquisition could occur with condomless vaginal or anal sex, 21.6% (219/1015) believed acquisition could occur with kissing, and 13% (129/1015) through insect bites (Fig. 2A). Regarding acquisition risk through condomless sex with PWH on effective treatment, 59.5% (604/1015) stated this was true, 20.5% (208/1015) stated this was not true, and 20% (203/1015) did not know (Fig. 2A).

**Fig. 3:**
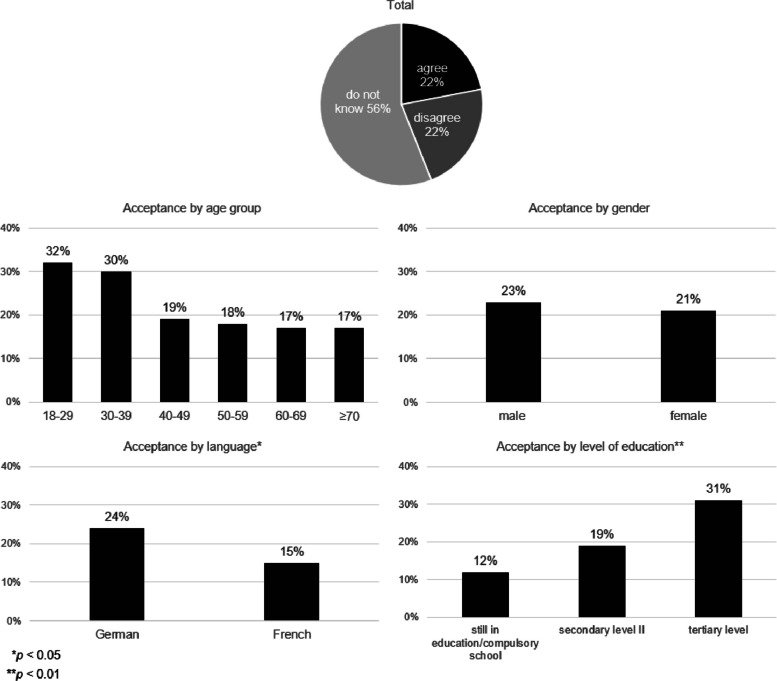
Acceptance of the U = U message of the total sample and by participant age group, gender, language and educational level (*N* = 1,015). Percentage values have been rounded up to the nearest whole number. Statistically significant differences are depicted with asterisks (* *p* < 0.05; ** *p* < 0.01)

When presented with the statement defining U = U (*If PWH have been on effective HIV therapy for at least six months (the virus is no longer detectable in the blood), they cannot transmit the virus through sex)*, 22% (223/1015) believed the statement to be true, 22.3% (226/1015) to be not true, and 55.8% (566/1015) did not know (Fig. 3). The acceptance of U = U as true was higher among those with higher education (*P* < 0.001) and among German speakers compared to French speakers (196/834 German speakers, 23.5%, believed U = U to be true compared to 26/177 of French speakers, 14.7%, *P* = 0.039) (Fig. 3).

For the other questions, 75.2% (763/1015) believed the statement that HIV was no longer a death sentence but a controllable chronic infection and 48% (487/1015) believed that women on effective HIV therapy with undetectable viral loads can now give birth to healthy children who are HIV-negative. Of the 12.6% (128/1015) of participants who had heard of PrEP, 54.7% (70/128) were able to identify the definition of PrEP as true.

### Attitudes towards PWH and HIV-stigma sum score

**Fig. 4:**
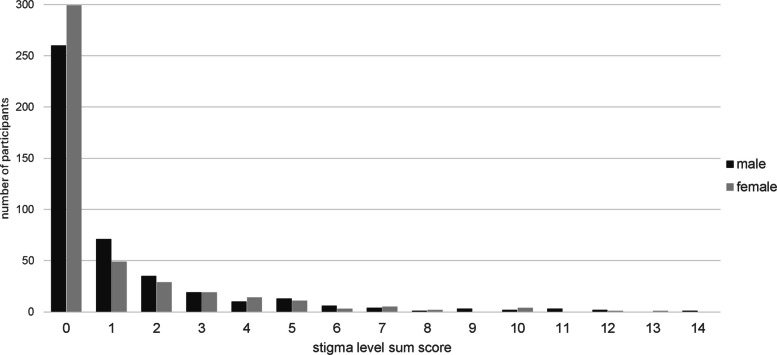
HIV-stigma sum scores among participants (430 men and 437 women). Sum scores were calculated from a subset of questions on attitudes towards people with HIV as listed in Table 1, excluding ‘don’t know’ responses

Most participants (74.6%, 758/1015) did not believe that PWH were a threat to society and 90.8% (922/1015) felt that PWH could be engaged in regular employment. Participants were generally comfortable with social contact with PWH except for marrying a PWH (23%, 234/1015) or having sex with PWH (13%, 132/1015) (Fig. 2B). The median HIV-stigma sum score was 2.88 (0.95;4.81). Excluding the 146 participants (of 1015, 14.4%) answering ‘don’t know’ to the questions in Table 1, 90.1% (783/869) of participants had low HIV-stigma sum scores (0—3), 9% (78/869) had moderate scores (4—10) and 0.9% (8/869) had high scores (11—14). Higher scores were associated with lower education level (*P* = 0.017) and male gender (*P* = 0.03). The spread of HIV-stigma sum scores by gender is shown in Fig. 4.

## Discussion

In this survey of 1015 members of the public in Switzerland, although 76% of participants stated they felt informed about HIV, only 22% agreed with the U = U message, and misconceptions regarding HIV acquisition were observed, including acquisition through kissing (22%), insect bites (13%), and shared use of everyday objects (8%). Fewer than half had ever been tested for HIV. Stigmatising attitudes towards PWH were overall low. Lower knowledge and higher stigmatising attitudes were associated with lower education level.

This is the first survey conducted on HIV knowledge and attitudes in the general public in Switzerland. In the United Kingdom, the National AIDS Trust examined public perception and knowledge of HIV among 3002 representative members of the population [9]. In this survey, in which the proportion of respondents living in rural versus non-rural areas was similar to our Swiss sample, 16% of respondents agreed with the U = U statement (defined as ‘zero risk’ of transmission) and 26% agreed with the definition of PrEP. Fewer respondents stated they would feel comfortable having a neighbour living with HIV (69%) or having sex with a PWH (9%) compared to our survey [9]. Another recent survey was conducted in Canada among 2681 sexual and gender minority men recruited from 16 LGBTQ2S + pride festivals. Compared to our survey, the participants in the Canadian study were younger, mean age 34.7 years old, 82.4% identified as being gay (our questionnaire did not ask about sexual orientation) and 7.1% were PWH compared to 0.6% in our survey. In this study, 73% of participants were aware of U = U [23].

The low awareness of U = U in our survey is perhaps not surprising given that communicating U = U even between healthcare providers (HCPs) and PWH is not universal, and that U = U is not always listed as a standard of care in national treatment guidelines [5, 24–26]. In a 25-country survey of 2389 PWH on treatment, Okoli et al. reported that, overall, 66.5% of PWH surveyed had ever discussed U = U with their HCP. In the Okoli et al. study, the figure for PWH having discussed U = U with their HCPs in Switzerland was higher at 87.3% (48/55 PWH) [26]. The authors observed that discussing U = U with an HCP was associated with improved health outcomes among PWH and increased HIV status sharing [26]. Although communicating U = U has a beneficial effect on health outcomes among PWH [26], a discretionary approach has been observed among HCPs, for example, restricting the discussion to PWH with undetectable, but not detectable, plasma viral loads, and thus potentially engendering enacted stigma [5].

One difficulty observed in communicating the U = U message is the concept of ‘zero risk’. Grace et al. conducted a qualitative study among 18 providers of sexual health services (sexual health nurses, public health workers, physicians, frontline workers, clinic counsellors, and sexual health educators) and reported that the notion of ‘zero risk’ with U = U was difficult to communicate because public health messages usually have some margin of error [24]. This was also observed among 270 British HCPs discussing U = U with PWH, where 37.2% described the risk of HIV acquisition as ‘zero’, while others described the risk as ‘extremely low’, ‘next to zero’, ‘virtually impossible’ or ‘negligible’ [25]. The difficulty of ‘zero risk’ has also been observed among members of the public. In a focus group organised within the National AIDS Trust survey described above, the concept of ‘zero risk’ was difficult to accept, as participants expressed that there is ‘no such thing’ as zero risk [9]. In our survey, we asked if it was true that transmission ‘cannot’ occur, without mentioning ‘zero risk’, and yet only 22% of participants agreed. Another way of presenting the U = U message is to frame it in terms of ‘protection’ rather than ‘risk’. Coyne et al. examined this within a nationally representative sample of 700 British residents. The authors reported a higher understanding of U = U following a protection-framed message and that this was associated with lower post-test HIV-stigma [27]. For the well-being of PWH and to reduce HIV-stigma, it is vital in parallel to optimise communication of U = U.

In our survey, the only consistent association with low HIV knowledge and stigmatising attitudes was lower education. Whilst a lower percentage of French-speaking participants believed that U = U was true compared to German-speakers, numbers in the former group were low. Equally, the higher HIV-stigma scores among men, whilst significant, related to higher scores within the low to moderate HIV-stigma range. Against the low knowledge of U = U and misconceptions regarding HIV acquisition observed in our survey, the sources of HIV information reported by over a third of participants were journalistic media, school/education and medical staff. Identifying these sources can guide future interventions to enhance HIV knowledge and address HIV-stigma.

Our survey has limitations. The recruitment method meant that participants were people living in Switzerland who had registered with Bilendi. Because respondents were recruited using a non‑probability, quota‑based panel, inclusion probabilities are unknown and formal sampling error cannot be estimated. For example, participants opt in, potentially favouring people with higher survey familiarity, greater topic interest and/or stronger opinions. While demographic quotas aim to optimise alignment with the general population, unobserved selection biases may remain as quotas control only observed variables. Unobserved, and therefore unquantifiable, drivers such as health status, cognitive ability, motivation, trust in institutions, media consumption, prior experience with the topic may have influenced questionnaire responses. As a result, estimates should be interpreted as descriptive rather than fully generalisable to the greater Swiss population. We also did not examine awareness of post-exposure prophylaxis (PEP) and so do not have this benchmark for future studies. Finally, participants may have presented less stigmatising attitudes through social desirability bias. Against these limitations, this is the first survey examining HIV knowledge in the general public in Switzerland and it reveals knowledge gaps and means by which these can be addressed.

In conclusion, although 76% of participants felt informed about HIV, only 22% agreed with the U = U message. Misconceptions regarding HIV acquisition risk were observed. High HIV-stigma scores were observed among a minority of participants and were associated with lower education. Tailored awareness campaigns using journalistic media and education in schools could enhance HIV knowledge, disseminate the U = U message and address societal HIV-stigma. Whilst such campaigns would contribute to bridging knowledge gaps regarding U = U and PrEP, qualitative studies are also necessary, to complement the questionnaire used in this study and enable a more detailed understanding of HIV knowledge and attitudes in the general population.

## Supplementary Information

Supplementary Material 1: Supplementary material S1. CHERRIES checklist for reporting results of web surveys.

Supplementary Material 2: Supplementary material S2. English translation of survey questionnaire.

Supplementary Material 3: Supplementary material S3. Sources of information about HIV (multiple responses possible).

## Data Availability

Data from this survey are not available to third parties.

## References

[1] Vernazza P, Hirschel B, Bernasconi E, Flepp M. HIV transmission under highly active antiretroviral therapy. Lancet. 2008;372:1806–7. 10.1016/S0140-6736(08)61753-5. (author reply 1807). 19027479 10.1016/S0140-6736(08)61753-5

[2] Prevention Access Campaign Consensus statement. Risk of sexual transmission of HIV from a person living with HIV who has an undetectable viral load. Available at https://www.preventionaccess.org/consensus.

[3] Cohen MS, et al. Antiretroviral therapy for the prevention of HIV-1 transmission. N Engl J Med. 2016;375:830–9. 27424812 10.1056/NEJMoa1600693 PMC5049503

[4] Rodger AJ, et al. Sexual activity without condoms and risk of HIV transmission in serodifferent couples when the HIV-positive partner is using suppressive antiretroviral therapy. JAMA. 2016;316:171–81. 10.1001/jama.2016.5148. 27404185 10.1001/jama.2016.5148

[5] Calabrese SK, Mayer KH. Stigma impedes HIV prevention by stifling patient-provider communication about U = U. J Int AIDS Soc. 2020;23:e25559. 10.1002/jia2.25559. 32686324 10.1002/jia2.25559 PMC7369401

[6] Ford OG, Rufurwadzo TG, Richman B, Green I, Alesi J. Adopting U = U to end stigma and discrimination. J Int AIDS Soc. 2022;25:e25891. 10.1002/jia2.25891. 35229483 10.1002/jia2.25891 PMC8886179

[7] Mendez-Lopez A, et al. Knowledge about biomedical HIV prevention among healthcare workers: a cross-sectional study in Europe and Central Asia. HIV Med. 2025. 10.1111/hiv.70048. 40390383 10.1111/hiv.70048

[8] Cobos Manuel I, et al. Stigma and HIV: relevant for everyone. Rev Med Suisse. 2020;16:744–8. 32301309

[9] National AIDS Trust. HIV: Public knowledge and attitudes. 2021. https://fasttrackcities.london/wp-content/uploads/2021/07/21033-NAT-Polling-Report-013S.pdf.

[10] Kampouri E, et al. Prevalence of HIV-related stigma among people with HIV in Switzerland: addressing the elephant in the room. AIDS. 2024. 10.1097/QAD.0000000000003983. 39051627 10.1097/QAD.0000000000003983 PMC11424058

[11] Gilles I, et al. Navigating HIV-related stigma in Switzerland: a qualitative study. Int J Public Health. 2024;69:1606333. 10.3389/ijph.2024.1606333. 38737988 10.3389/ijph.2024.1606333 PMC11082645

[12] Le Saux C, et al. Once HIV knowledge is addressed: HIV-stigma from the perspective of healthcare professionals working in HIV facilities in French-speaking Switzerland. J Int AIDS Soc. 2024;27:227–8. 10.3389/ijph.2026.1609379 PMC13065731 41969931

[13] Stigma: survey of people living with HIV. Monitoring implementation of the Dublin Declaration on partnership to fight HIV/AIDS in Europe and Central Asia: 2022 progress report. 2023. https://www.ecdc.europa.eu/sites/default/files/documents/hiv-stigma-survey-monitoring-dublin-declaration.pdf.

[14] UNAIDS. Global data on HIV epidemiology and response 2023. Switzerland. https://aidsinfo.unaids.org/. Accessed 20 Feb 2026.

[15] Federal Office of Public Health. Statistics and analyses concerning HIV/STI. 2023. https://www.bag.admin.ch/fr/statistiques-et-analyses-concernant-vih-ist. Accessed 20 Feb 2026.

[16] SwissPrEPared. https://www.swissprepared.ch/en/. Accessed 20 Feb 2026.

[17] Hovaguimian F, et al. Participation, retention and uptake in a multicentre pre-exposure prophylaxis cohort using online, smartphone-compatible data collection. HIV Med. 2022;23:146–58. 10.1111/hiv.13175. 34605153 10.1111/hiv.13175 PMC9292805

[18] Federal Office of Public Health. Infections sexuellement transmissibles et hépatites B/C en Suisse et au Liechtenstein: évaluation épidémiologique. Bull OFSP. 2023;48:8–11.

[19] Swiss Ethics Committees on research involving humans. Quality assurance, or research subject to approval? https://swissethics.ch/assets/pos_papiere_leitfaden/191223_abgrenzung-qualitatssicherung-von-forschung_finalisierte-version_de_en.pdf. Last accessed 21 July 2025.

[20] Eysenbach G. Improving the quality of Web surveys: the Checklist for Reporting Results of Internet E-Surveys (CHERRIES). J Med Internet Res. 2004;6:e34. 10.2196/jmir.6.3.e34. 15471760 10.2196/jmir.6.3.e34 PMC1550605

[21] UNAIDS Terminology Guidelines 2024 https://www.unaids.org/en/resources/documents/2024/terminology_guidelines Accessed 12 Mar 2026.

[22] Joint United Nations Programme on HIV/AIDS. Global AIDS Monitoring 2025. Indicators and questions for monitoring progress on the 2021 Political Declaration on HIV and AIDS. 2025. https://www.unaids.org/sites/default/files/media_asset/global-aids-monitoring_en.pdf.

[23] Card KG, et al. Who knows about U = U? Social positionality and knowledge about the (un)transmissibility of HIV from people with undetectable viral loads. AIDS Care. 2022;34:753–61. 10.1080/09540121.2021.1902928. 33739198 10.1080/09540121.2021.1902928

[24] Grace D, et al. Challenges to communicating the undetectable equals untransmittable (U=U) HIV prevention message: healthcare provider perspectives. PLoS ONE. 2022;17:e0271607. 10.1371/journal.pone.0271607. 35862361 10.1371/journal.pone.0271607 PMC9302742

[25] Gupta N, Gilleece Y, Orkin C. Implementing U=U in clinical practice: results of a British HIV association members survey. Sex Transm Infect. 2021;97:619–20. 10.1136/sextrans-2020-054462. 32139498 10.1136/sextrans-2020-054462

[26] Okoli C, et al. Undetectable equals untransmittable (U = U): awareness and associations with health outcomes among people living with HIV in 25 countries. Sex Transm Infect. 2021;97:18–26. 10.1136/sextrans-2020-054551. 32732335 10.1136/sextrans-2020-054551 PMC7841488

[27] Coyne R, Walsh JC, Noone C. Awareness, understanding and HIV stigma in response to undetectable = untransmittable messages: findings from a nationally representative sample in the United Kingdom. AIDS Behav. 2022;26:3818–26. 10.1007/s10461-022-03710-9. 35687191 10.1007/s10461-022-03710-9 PMC9640424

