# Supplementary material for: Public knowledge and attitudes towards HIV and people with HIV in Switzerland: results of a national survey
Source: BMC Public Health. 2026 May 18;26:2133. doi: 10.1186/s12889-026-27629-1 (PMC13359419; doi:10.1186/s12889-026-27629-1)
Supplement: Supplementary file 1 — Supplementary Material 1: Supplementary material S1. CHERRIES checklist for reporting results of web surveys. [file 12889_2026_27629_MOESM1_ESM.docx]

**Supplementary material S1: CHERRIES Checklist for Online Questionnaires**

| Study Design: Describe target population & sample frame. Is the sample a convenience sample? | It is a non‑probability, quota‑based panel sampling / convenience sampling with demographic quotas. Quotas introduced to increase representation of the Swiss population: age, gender, region, and level of education |
| --- | --- |
| IRB Approval: Has the study been approved by an IRB? | Not applicable |
| Informed consent:  Describe the informed consent process. Where were the participants told the length of time of the survey, which data were stored & where/for how long, who the investigator was, purpose of the study? | The introductory paragraph at the start of the survey, including approximate length of time required   - Description of the questionnaire purpose - Participants informed that no identifying data will be collected   Participants were also informed that participating in the survey will imply their consent for their answers to be used for research. |
| Data protection: If any personal information was collected or stored, describe what mechanisms were used to protect unauthorised access. | No personal information was collected or stored. |
| Development and testing | The survey was reviewed for content validity by three people living with HIV (two of whom represent community organisations) and two HIV physicians |
| Open survey versus closed survey | This is a closed survey.  Participants had to enter a password to access the survey. |
| Contact mode:  Indicate whether or not the initial contact with the potential participants was made on the Internet. | Participants were already part of an existing panel |
| Advertising the survey:  How/where was the survey announced or advertised? It is important to know the wording of the announcement as it will heavily influence who chooses to participate. | Bilendi recruited exclusively from its own online panel, with email notification and via its own platform, where registered participants are shown the surveys assigned to them. No recruitment took place via social media.  <https://www.bilendi.us/static/quality> |
| Web/Email:  State the type of e-survey (e.g. one posted on a Web site or one sent out through e-mail). If it is an e-mail survey, were the responses entered manually into a database, or was there an automatic method for capturing responses? | Web-based survey |
| Context:  Describe the Web site (for mailing list/newsgroup) in which the survey was posted. | Surveys are being performed on the Bilendi proprietary platform. Also see: <https://www.bilendi.us/static/quality> |
| Mandatory/voluntary:  Was it a mandatory survey to be filled in by every visitor who wanted to enter the Web site, or was it a voluntary survey? | Voluntary |
| Incentives:  Were any incentives offered (e.g., monetary, prizes, or non-monetary incentives such as an offer to provide the survey results)? | We incentivised our participants in Switzerland with money or points. The amount of compensation depends on the length of the questionnaire. For this survey, participants received CHF 1.60 or the equivalent in points (40 points). |
| Time/Date:  In what timeframe were the data collected? | 5-11 October, 2023 |
| Randomisation of items:  To prevent biases items can be randomised or alternated. | The items were not randomised |
| Adaptive questioning:  Use adaptive questioning (certain items, or only conditionally displayed based on responses to other items) to reduce number and complexity of the questions. | Adaptive Question:  Q15 (“PrEP is …”) was skipped, if the following answer was selected in Q14: “I have never heard about PrEP”. |
| Number of Items:  What was the number of questionnaire items per page? The number of items is an important factor for the completion rate. | 1 item per page |
| Number of Screens:  Over how many pages was the questionnaire distributed? The number of items is an important factor for the completion rate. | 26 (1 Introduction Text, 24 Question Items, 1 Explanatory Text at the end about U=U, transmission routes, PrEP) |
| Completeness check:  Were consistency or completeness checks before questionnaire submission? An alternative is to check for completeness after the questionnaire has been submitted (and highlight mandatory items). | Yes, completeness checks at single item level |
| Unique site visitor | Participation only upon invitation (quota-based random sampling) |
| View rate (Ratio of unique survey visitors/unique site visitors) | N/A |
| Participation rate | Of 1795 potential participants approached, 1015 completed the questionnaire |
| Completion rate (Ratio of users who finished the survey/users who agreed to participate): This is only relevant if there is a separate “informed consent” page or if the survey goes over several pages. This is a measure for attrition. | Not calculated |
| Cookies used:  Indicate whether cookies were used to assign a unique user identifier to each client computer. | Registration (non-open survey): Unique user identifier provided by Bilendi platform account credentials and quota-based random invitations by Email. |
| IP check:  Indicate whether the IP address of the client computer was used to identify potential duplicate entries from the same user. |  |
| Log file analysis:  Indicate whether other techniques to analyse the log file for identification of multiple entries were used. If so, please describe. | Registration (non-open survey): Unique user identifier provided by Bilendi platform account credentials and quota-based random invitations by Email. |
| Registration:  In “closed” (non-open) surveys, users need to login first and it is easier to prevent duplicate entries from the same user. |  |
| Handling of incomplete questionnaires:  Were only completed questionnaires analysed? | Only completed questionnaires are analysed |
| Statistical correction:  Indicate whether any methods such as weighting of items or propensity scores have been used to adjust for the non-representative sample; if so, please describe the methods. | Introduction of quotas: age, gender, region, and level of education to represent the Swiss population |
