# Supplementary material for: Public knowledge and attitudes towards HIV and people with HIV in Switzerland: results of a national survey
Source: BMC Public Health. 2026 May 18;26:2133. doi: 10.1186/s12889-026-27629-1 (PMC13359419; doi:10.1186/s12889-026-27629-1)
Supplement: Supplementary file 2 — Supplementary Material 2: Supplementary material S2. English translation of survey questionnaire. [file 12889_2026_27629_MOESM2_ESM.docx]

**Supplementary material S2. Survey questionnaire: Public opinion on HIV**

**Section 1:** General knowledge and attitudes regarding health-related topics

Q1. How well do you think you are informed about health-related topics in general (various diseases and options for prevention and/or treatment)?

*[Simple answer]*

| 1) | Very well informed |
| --- | --- |
| 2) | Quite well informed |
| 3) | Not so well informed |
| 4) | Not up to date at all |

Q2. How well informed are you about the individual infections/ diseases/ health conditions listed?

*[simple matrix] [rotation]*

|  |  | **Very well informed** | **Sufficiently well informed** | **Not so well informed** | **Not informed at all** |
| --- | --- | --- | --- | --- | --- |
| 1) | Diabetes | (1) | (2) | (3) | (4) |
| 2) | Cancer | (1) | (2) | (3) | (4) |
| 3) | HIV/AIDS | (1) | (2) | (3) | (4) |
| 4) | Coronavirus (COVID-19) | (1) | (2) | (3) | (4) |
| 5) | Hypertension | (1) | (2) | (3) | (4) |
| 6) | Syphilis or chlamydia | (1) | (2) | (3) | (4) |
| 7) | Hepatitis C | (1) | (2) | (3) | (4) |

Q3. How dangerous or life-threatening do you consider the following infections/ diseases/ health conditions to be?

*[Simple matrix] [Rotation as Q2]*

|  |  | **Very**  **threatening** | **Quite threatening** | **Less threatening** | **Not**  **threatening** | *I don’t know* |
| --- | --- | --- | --- | --- | --- | --- |
| 1) | Diabetes | (1) | (2) | (3) | (4) | *(99)* |
| 2) | Cancer | (1) | (2) | (3) | (4) | *(99)* |
| 3) | HIV/AIDS | (1) | (2) | (3) | (4) | *(99)* |
| 4) | Coronavirus (COVID-19) | (1) | (2) | (3) | (4) | *(99)* |
| 5) | Hypertension | (1) | (2) | (3) | (4) | *(99)* |
| 6) | Syphilis or chlamydia | (1) | (2) | (3) | (4) | *(99)* |
| 7) | Hepatitis C | (1) | (2) | (3) | (4) | *(99)* |

Q4. On a scale of 1 to 10, please rate whether there have been medical advances in the treatment of the following infections/ diseases/ health conditions in the last ten years.

A value of 10 means significant progress and a value of 1 means no progress in treatment.

*[Simple matrix] [Rotation as Q2]*

|  |  | *10 = significant* progress; 1 *= no* progress | *I don’t know* |
| --- | --- | --- | --- |
| 1) | Diabetes | ➉ ➈ ➇ ➆ ➅ ➄ ➃ ➂ ➁ ➀ | 🞎 |
| 2) | Cancer | ➉ ➈ ➇ ➆ ➅ ➄ ➃ ➂ ➁ ➀ | 🞎 |
| 3) | HIV/AIDS | ➉ ➈ ➇ ➆ ➅ ➄ ➃ ➂ ➁ ➀ | 🞎 |
| 4) | Coronavirus (COVID-19) | ➉ ➈ ➇ ➆ ➅ ➄ ➃ ➂ ➁ ➀ | 🞎 |
| 5) | Hypertension | ➉ ➈ ➇ ➆ ➅ ➄ ➃ ➂ ➁ ➀ | 🞎 |
| 6) | Syphilis or chlamydia | ➉ ➈ ➇ ➆ ➅ ➄ ➃ ➂ ➁ ➀ | 🞎 |
| 7) | Hepatitis C | ➉ ➈ ➇ ➆ ➅ ➄ ➃ ➂ ➁ ➀ | 🞎 |

**Section 2: Focus on HIV/AIDS: information sources and personal experience**

**[If in Q2- 3 HIV response 1-3]**

Q5. You indicated that you have some level of knowledge about HIV or AIDS. How did you obtain information?

*[Rotation] [Multiple answers possible] [1-9 not combinable with 10] [9 = + open entry]*

| 1) | Family and/or friend(s) |
| --- | --- |
| 2) | Sexual partner(s) |
| 3) | School/training (eg, sex education) |
| 4) | Medical professionals (doctors, nurses, etc.) |
| 5) | LOVE LIFE campaign, Federal Office of Public Health |
| 6) | AIDS support services, sexual health services |
| 7) | Journalistic media (television, newspaper, websites) |
| 8) | Social media (Facebook, Instagram, etc) |
| 9) | Other? ______ |
| *10)* | *I can’t remember* |

Q6. Please think about any information about HIV or AIDS that you have seen, heard or read in the last 6 months. Was this information more positive or more negative about HIV/AIDS or people living with HIV?

*[Simple answer]*

| 1) | positive |
| --- | --- |
| 2) | rather positive |
| 3) | neutral |
| 4) | rather negative |
| *5)* | *negative* |
| *6)* | *I have not noticed any information on HIV/ AIDS in the last 6 months* |
| *7)* | *I can’t remember* |

Q7. Do you personally know one or more people living with HIV?

*[Rotation] [Multiple answers possible] [1-6 with 7+8 not combinable] [6 = + open entry]*

| 1) | Yes, myself |
| --- | --- |
| 2) | Yes, parents, children, siblings |
| 3) | Yes, spouse, partner |
| 3) | Yes, other relatives |
| 4) | Yes, friend, colleague |
| 5) | Yes, simple acquaintances |
| 6) | Yes, other: ____________ |
| *7)* | *None of the above* |
| *8)* | *I don’t know* |

Q8. Have you personally ever had an HIV test? If so, how many years ago was the last test?

*[Simple answer]*

| 1) | Yes, within the last 12 months |
| --- | --- |
| 2) | Yes, 2-5 years ago |
| 3) | Yes, more than 5 years ago |
| 4) | No, never |
| *5)* | *I can’t remember* |

**Section 3: Knowledge and attitudes towards HIV/AIDS**

Q9. To what extent would you say that people with HIV present a danger to society?

*[Simple answer]*

| 1) | No danger |
| --- | --- |
| 2) | Barely |
| 3) | Often |
| 4) | Always |
| *5)* | *I don’t know* |

Q10. And to what extent do you believe that people with HIV or AIDS can engage in regular employment?

*[Simple answer]*

| 1) | Never |
| --- | --- |
| 2) | Rarely |
| 3) | Often |
| 4) | Always |
| *5)* | *I don’t know* |

Q11. Please read the following statements carefully and answer them exactly as you feel, as honestly as possible. Would you mind ...?

*[simple matrix] [rotation]*

|  |  | I don’t mind at all | I don’t mind so much | I mind a little | I would definitely not do that | *I don’t know* |
| --- | --- | --- | --- | --- | --- | --- |
| 1) | ... sitting next to a person with HIV? | (4) | (3) | (2) | (1) | *(99)* |
| 2) | ... marrying a person with HIV? | (4) | (3) | (2) | (1) | *(99)* |
| 3) | … lending something to a person with HIV? | (4) | (3) | (2) | (1) | *(99)* |
| 4) | ... starting a friendship with a person with HIV? | (4) | (3) | (2) | (1) | *(99)* |
| 5) | ... working with a person with HIV? | (4) | (3) | (2) | (1) | *(99)* |
| 6) | ... living next to a person with HIV? | (4) | (3) | (2) | (1) | *(99)* |
| 7) | ... having sex with a person with HIV? | (4) | (3) | (2) | (1) | *(99)* |

Q12. According to your knowledge, how can HIV be transmitted? *(If you don’t know, choose option "I don’t know".)*

*[simple matrix] [rotation]*

|  |  | True | Not true | *I don’t know* |
| --- | --- | --- | --- | --- |
| 1) | Through insect bites | (1) | (2) | *(99)* |
| 2) | Through sharing of syringes / injection needles | (1) | (2) | *(99)* |
| 3) | Through sharing everyday objects, such as glasses / dishes / cutlery | (1) | (2) | *(99)* |
| 4) | Through sharing a toilet | (1) | (2) | *(99)* |
| 5) | Through non-professional tattoos or piercings | (1) | (2) | *(99)* |
| 6) | Through shaking hands with a person who has HIV | (1) | (2) | *(99)* |
| 7) | Through hugging a person who has HIV | (1) | (2) | *(99)* |
| 8) | Through kissing a person who has HIV |  |  |  |
| 9) | Through vaginal or anal sex without a condom with a person who has HIV | (1) | (2) | *(99)* |
| 10) | Through sex with a person who has HIV and is receiving effective treatment | (1) | (2) | *(99)* |

Q13. Based on your knowledge or previous experience, please indicate whether you agree or disagree with the following statements. *(If you can’t answer a sentence, choose option, ‘I don’t know’.)*

*[Single matrix or slider] [Rotation]*

|  |  | True | Not true | *I don’t know* |
| --- | --- | --- | --- | --- |
| 1) | If people with HIV have been on effective HIV therapy for at least 6 months (so the virus is no longer detectable in blood), they cannot transmit the virus to their partners through sex. | (1) | (2) | *(99)* |
| 2) | Thanks to modern HIV therapy, HIV is no longer a death sentence but a controllable chronic viral infection. | (1) | (2) | *(99)* |
| 3) | Thanks to effective HIV therapy (where the virus is no longer detectable in blood), women with HIV can now give birth to healthy children who are not HIV-positive. | (1) | (2) | *(99)* |

Q14. Have you ever heard the term "PrEP" - and if so, from whom?

*[Multiple answers possible, 5 not combinable with 1-4]*

| 1) | Yes, from a doctor / a physician |
| --- | --- |
| 2) | Yes, in a sexual health centre |
| 3) | Yes, from a sexual partner |
| 4) | Yes, read/heard about it in the media |
| *5)* | *I have never heard of PrEP* |

*Skip if Q14 (5) = “I have never heard of"*

Q15. Which of these statements best describes “PrEP” to your knowledge? PrEP is …

*[Simple answer]*

| 1) | ... emergency treatment after an HIV acquisition risk situation |
| --- | --- |
| 2) | ...a preventive medication that protects against HIV as reliably as a condom |
| 3) | ...a new, innovative therapy for people with HIV |
| 4) | *I don’t know* |

**Section 4: Demography**

Thank you for taking the time to participate in this survey!

Finally, we would like to ask you for some information for statistical purposes.

| Q16. | Please enter your gender**:** |
| --- | --- |

| 1. | Male | 🞏_1_ |
| --- | --- | --- |
| 2. | Female | 🞏_2_ |
| 3. | Divers | 🞏_3_ |

| Q17. | Please indicate which age group applies to you: |
| --- | --- |

| 1. | Up to 18 years | 🞏_1_ |  | 7. | 51 - 55 years | 🞏_7_ |
| --- | --- | --- | --- | --- | --- | --- |
| 2. | 19 - 25 years | 🞏_2_ |  | 8. | 56 - 60 years | 🞏_8_ |
| 3. | 26 - 35 years | 🞏_3_ |  | 9. | 61 - 65 years | 🞏_9_ |
| 4. | 36 - 40 years | 🞏_4_ |  | 10. | 66 - 70 years | 🞏_10_ |
| 5. | 41 - 45 years | 🞏_5_ |  | 11. | 71 - 75 years | 🞏_11_ |
| 6. | 46 - 50 years | 🞏_6_ |  | 12. | Older than 75 years | 🞏_12_ |

| Q 18. | Please specify the canton in which you live: |
| --- | --- |

| Aargau | ⬜ _1_ |  | Nidwalden | ⬜ _14_ |
| --- | --- | --- | --- | --- |
| Appenzell (outer) | ⬜ _2_ |  | Obwalden | ⬜ _15_ |
| Appenzell (inner) | ⬜ _3_ |  | St Gallen | ⬜ _16_ |
| Basel-Countryside | ⬜ _4_ |  | Schaffhausen | ⬜ _17_ |
| Basel-City | ⬜ _5_ |  | Schwyz | ⬜ _18_ |
| Bern | ⬜ _6_ |  | Solothurn | ⬜ _19_ |
| Freiburg | ⬜ _7_ |  | Thurgau | ⬜ _20_ |
| Geneva | ⬜ _8_ |  | Ticino | ⬜ _21_ |
| Glarus | ⬜ _9_ |  | Uri | ⬜ _22_ |
| Grindelwald | ⬜ _10_ |  | Vaud | ⬜ _23_ |
| Jura | ⬜ _11_ |  | Valais | ⬜ _24_ |
| Lucerne | ⬜ _12_ |  | Zug | ⬜ _25_ |
| Neuchâtel | ⬜ _13_ |  | Zurich | ⬜ _26_ |

| Q19. | How big is the place where you live? |
| --- | --- |

| 1. | More than 50,000 inhabitants | 🞏_1_ |
| --- | --- | --- |
| 2. | 10,000 - 50,000 inhabitants | 🞏_2_ |
| 3. | Fewer than 10,000 inhabitants | 🞏_3_ |

| Q20. | Do you live...? |
| --- | --- |

| 1. | In a city |
| --- | --- |
| 2. | Close to a city |
| 3. | Far from a city |

| Q21. | Please indicate your civil status: |
| --- | --- |

| 1. | Single |
| --- | --- |
| 2. | Married / registered civil partnership / in a relationship |
| 3. | Divorced / registered civil partnership annulled |
| 4. | Widowed / registered civil partner deceased |

| Q 22. | Please enter your highest education level. |
| --- | --- |

| 1. | Still in school / training |
| --- | --- |
| 2. | Completed compulsory school |
| 3. | Completed compulsory school with apprenticeship |
| 4. | Vocational secondary school (eg, business or technical school) without *Matura* |
| 5. | Secondary school or vocational school (eg, commercial academy) with *Matura* |
| 6. | Completed university or federal technical college |
| *7.* | *I do not want to give any information* |

| Q 23. | **Which of the following categories best describes your employment status?** |
| --- | --- |

| 1. | Full-time employed |
| --- | --- |
| 2. | Part-time employed |
| 3. | Self-employed / freelance |
| 4. | Student |
| 5. | Retired |
| 6. | Unemployed / jobseeker |
| 7. | Parental leave / housewife / househusband |
| 8. | Short-time work |
| 9. | On sickness benefit |
| *10.* | *I do not want to give any information* |

| Q24. | What is your approximate monthly household income after deduction of taxes & social security contributions? |
| --- | --- |

| 1. | Up to CHF 1,000 |
| --- | --- |
| 2. | 1.000,- to under 2.000,- CHF |
| 3. | 2.000,- to under 3.000,- CHF |
| 4. | 3.000,- to under 4.000,- CHF |
| 5. | 4.000,- to under 5.000,- CHF |
| 6. | 5.000,- to under 6.000,- CHF |
| 7. | 6.000,- CHF and more |
| 8. | *I do not want to give any information* |
