# Supplementary figures and images for: Public knowledge and attitudes towards HIV and people with HIV in Switzerland: results of a national survey
Source: BMC Public Health. 2026 May 18;26:2133. doi: 10.1186/s12889-026-27629-1 (PMC13359419; doi:10.1186/s12889-026-27629-1)

**Supplementary material S3: Sources of HIV knowledge**


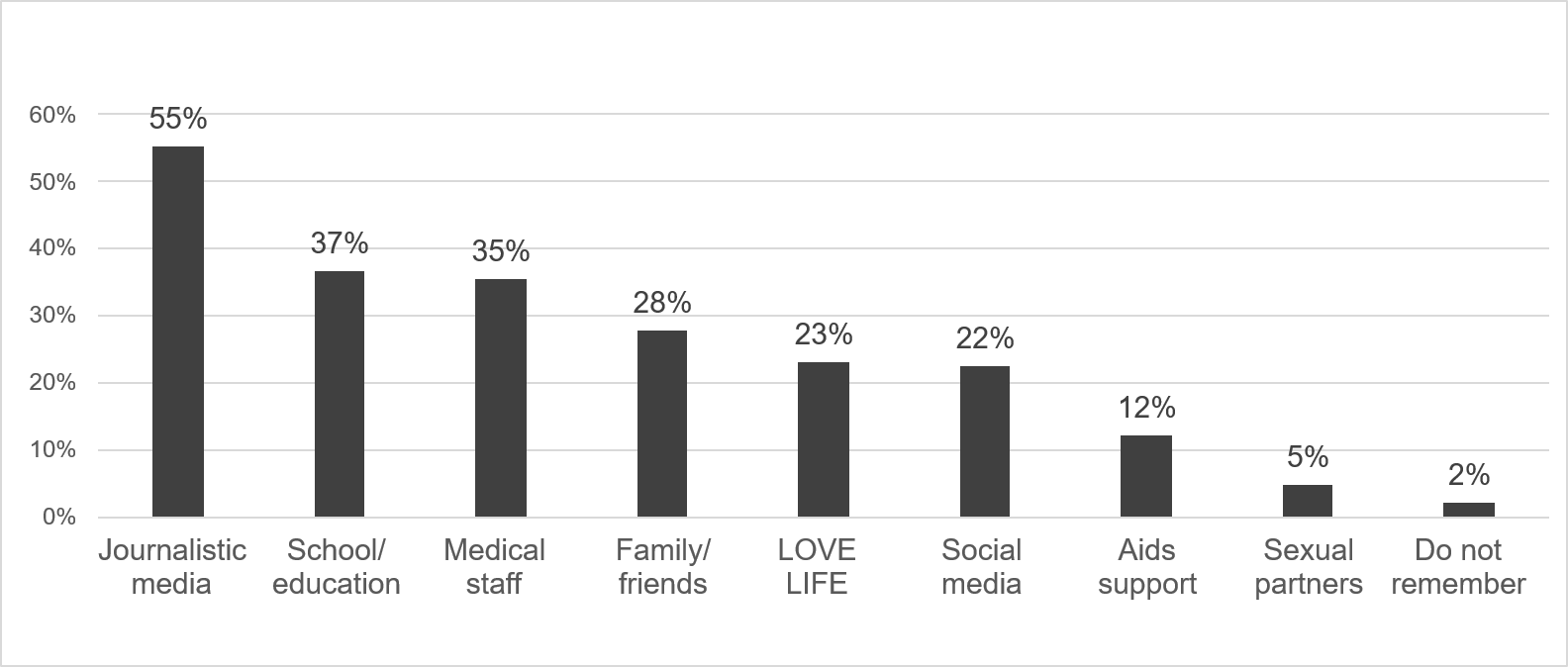

Supplement: Supplementary file 3 — Supplementary Material 3: Supplementary material S3. Sources of information about HIV (multiple responses possible). [file 12889_2026_27629_MOESM3_ESM.docx]
